# Ultra-Porous Nanocellulose Foams: A Facile and Scalable Fabrication Approach

**DOI:** 10.3390/nano9081142

**Published:** 2019-08-09

**Authors:** Carlo Antonini, Tingting Wu, Tanja Zimmermann, Abderrahmane Kherbeche, Marie-Jean Thoraval, Gustav Nyström, Thomas Geiger

**Affiliations:** 1 Cellulose and Wood Materials, Swiss Federal Laboratories for Materials Science and Technology (Empa), 8600 Dübendorf, Switzerland; 2 Department of Materials Science, University of Milano—Bicocca, 20126 Milano, Italy; 3 State Key Laboratory for Strength and Vibration of Mechanical Structures, Shaanxi Key Laboratory of Environment and Control for Flight Vehicle, International Center for Applied Mechanics, School of Aerospace, Xi’an Jiaotong University, Xi’an 710049, China

**Keywords:** cellulose nanomaterials, nanofibrils, foam, oil absorption, hydrophobicity, ice-templating, freeze-drying, freeze-thawing, ambient pressure drying, compound drops

## Abstract

Cellulose nanofibril foams are cellulose-based porous materials with outstanding mechanical properties, resulting from the high strength-to-weight ratio of nanofibrils. Here we report the development of an optimized fabrication process for highly porous cellulose foams, based on a well-controlled freeze-thawing-drying (FTD) process at ambient pressure. This process enables the fabrication of foams with ultra-high porosity, up to 99.4%, density of 10 mg/cm^3^, and liquid (such as oil) absorption capacity of 100 L/kg. The proposed approach is based on the ice-templating of nanocellulose suspension in water, followed by thawing in ethanol and drying at environmental pressures. As such, the proposed fabrication route overcomes one of the major bottle-necks of the classical freeze-drying approach, by eliminating the energy-demanding vacuum drying step required to avoid wet foam collapse upon drying. As a result, the process is simple, environmentally friendly, and easily scalable. Details of the foam development fabrication process and functionalization are thoroughly discussed, highlighting the main parameters affecting the process, e.g., the concentration of nanocellulose and additives used to control the ice nucleation. The foams are also characterized by mechanical tests and oil absorption measurements, which are used to assess the foam absorption capability as well as the foam porosity. Compound water-in-oil drop impact experiments are used to demonstrate the potential of immiscible liquid separation using cellulose foams.

## 1. Introduction

Cellulose nanofibrils (CNFs) are cellulose-based, biodegradable, renewable, and intrinsically amphiphilic materials, composed of high-aspect ratio nanofibrils, obtained from a fibrillation process of cellulose pulp. Due to the high strength-to-weight ratio of the nanofibrils, outstanding mechanical properties can be achieved using nanofibrils as building blocks [1,2,3,4]. As an example, cellulose nanopaper shows a remarkable increase in both strength and toughness as the size of the constituent cellulose fibers decreases [5]. Porous foams are attracting increasing attention for their potential in a wide variety of applications, where ultralow density and high surface areas are required [6,7], including thermal insulation [8], gas adsorption [9], energy storage [10,11], and selective liquid absorption for environmental remediation [12,13,14,15], for biofuel purification [16], and for ethanol extraction from an aqueous solution [17]. One commonly used technique to fabricate CNF foams is based on ice templating, through the well-known freeze-drying process. Ice templating has been tested with different type of materials, from ceramics to metals, polymers, and carbon materials, such as graphene or nanotubes, including their composites [18]. In the case of cellulose, a suspension of CNF in water is frozen and vacuum-dried to obtain dry foams [8,11,19]. The ice crystals formed during the freezing process provide the negative template for CNF to assemble and form the foam skeleton [20]. Afterwards, ice is directly removed though sublimation at low pressure, leaving a porous structure. This drying process avoids the intermediate water liquid state, which typically causes the foam structure to collapse upon liquid evaporation due to capillary forces. However, vacuum drying is a high energy-demanding step and currently represents a bottle-neck for the process scale-up needed for industrially relevant applications. As such, the first challenge is to develop new processes avoiding the vacuum-drying step, while achieving exceptional porosity, higher than 99%. In addition, achieving selective absorption of oils and hydrocarbons (e.g., to remediate water contaminations) requires tuning of the cellulose wettability. Several techniques to modify cellulose wetting from amphiphilic (hydrophilic/oleophilic) to hydrophobic/oleophilic have already been developed and presented, e.g., in [12,21,22,23]. However, simple foam wettability tuning, made possible through a facile, scalable and environmental-friendly approach, is still desirable.

To address the first challenge, i.e., avoiding the vacuum-drying step, we present the development and optimization of a straightforward freeze-thawing-drying (FTD) procedure, based on the use of urea as an additive to the CNF-water suspension and a solvent exchange step included to minimize foam collapse during drying. The positive effect of urea as an additive has been already presented in a previous paper by our group [24], in which light foams with a density of 30 mg/cm^3^ were fabricated. In the present paper, we address the specific goal of minimizing foam density, maximizing porosity, and, as consequence, liquid absorption capacity. Importantly, the whole fabrication process to prepare samples is also drastically reduced from 8–10 days to 8 h. The rapid production is achieved by the development of an optimized fabrication process, where the role of the CNF suspension concentration and a control of the freezing process for the ice templating has been systematically investigated. This method allows the production of mechanically stable, ultra-lightweight CNF structures without foam collapse using a facile thawing-drying procedure, with high porosity, up to 99.4%, and low density (10 mg/cm^3^) [25]. Values are comparable to those of freeze-dried foams, ranging from 5.6 to 60 mg/cm^3^ [7,8,12,19,21], which however require vacuum drying to sublimate ice and avoid foam collapse. In addition to the process development, we also identify and discuss the possible interaction mechanisms that enable stable foam drying in the CNF-water-urea based system. Finally, to demonstrate the potential use for selective oil absorption, foams were made superhydrophobic using alkylketenedymer (AKD), an organic compound, widely used to reduce hydrophilicity and control wetting in the paper industry [26]. The AKD-modified foams showed a characteristic superhydrophobic behavior, with rebound of impacting water drops. Also, by performing impact of water-in-oil compound drops we demonstrated the potential of using nanocellulose foams for separation of water, which rebounds after impact, from the oil absorbed by the foam.

## 2. Results and Discussion

**Figure 1:**
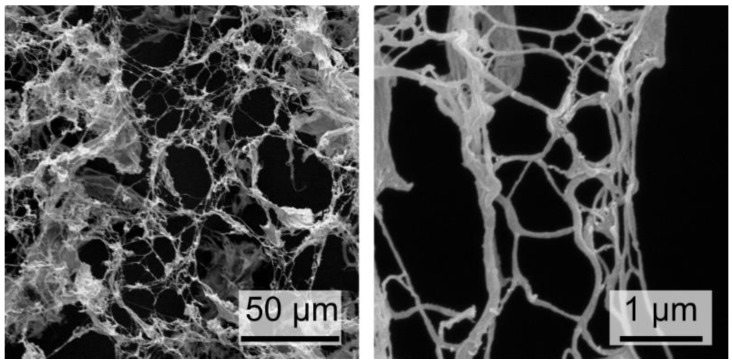
SEM images at different magnification of dry cellulose nanofibrils, after mechanical fibrillation using an ultra-fine friction grinder.

*Cellulose nanofibrils.* The starting CNF suspensions were produced from dry cellulose boards, which were swollen in water and mechanically ground using an ultra-fine friction grinder, as detailed in the Methods and Materials section. SEM images reported in Figure 1. visualize how the resulting cellulose nanofibril diameters span over a wide range, including diameters below 100 nm. The specific surface area, as measured by the BET (Brunauer-Emmett-Teller) method for the super-critically dried nanofibrils, is 200 m^2^/g, demonstrating the high degree of fiber fibrillation.

**Figure 2:**
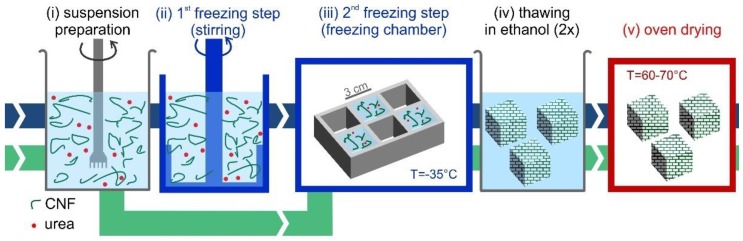
Schematic of the freezing-thawing-drying (FTD) foam fabrication process performed at environmental pressure. The stirred freezing process (blue path) consists of: (**i**) preparation and mixing of the Cellulose nanofibril (CNF) suspension in water with dissolved urea as an additive; (**ii**) first freezing step, inside an ice-cream machine, during which the suspension is partially frozen; (**iii**) second freezing step, during which sample freezing is completed in a silicone mold inside a freezer at −35 °C; (**iv**) thawing and washing of the sample in ethanol; and (**v**) oven drying. For the static freezing process (green path), the first freezing step is skipped, and the CNF suspension is frozen directly in the freezing chamber.

*Freeze-thawing-drying fabrication process.* A method based on the ice-templating of CNF suspensions, schematically shown in Figure 2, was developed and optimized to form stable CNF foams. CNF-water suspensions were initially prepared at room temperature by diluting the initial highly concentrated suspension, to reach CNF concentrations in the range 0.3 wt% to 3.0 wt%. Urea was pre-dissolved in water at 50 wt% concentration and added to the CNF suspension to dilute it to the same solid concentration as CNF. As detailed in Supplemental Information, preliminary optimizations tests showed that a 1:1 ratio of CNF-urea maximized oil absorption capacity. The CNF suspensions were frozen following two different freezing routes. In the first route, which is referred to as the “static freezing process” (green path in Figure 2), the CNF suspension was poured in pre-cooled silicone molds with cubic shapes (33 mL) and stored in a freezer for at least 3 h at −35 °C, to obtain completely frozen suspensions. In the second route, “stirred freezing process” (blue path in Figure 2), a commercial ice-cream machine “ICM” (Unold, mod. 48845) was used to cool down the CNF suspension temperature to the freezing point and initiate the freezing process under continuous stirring, to obtain a partially frozen suspension with homogeneous ice crystal distribution, similar to a sorbet. The CNF suspension was processed with the ice-cream machine in batches of 500 g with a process time *t*_sf_ ≈ 12 min (where “sf” stands for “stirred freezing”), measured from the moment when the freezing starts. Based on the heat transfer rate, it was estimated that within this time (*t*_sf_) 50% of water in the suspension froze.

The partially frozen suspension was then poured into silicone molds with cubic shapes (33 mL) and stored in a freezer for at least 3 h at −35 °C, to obtain completely frozen suspensions. In this work, the freezing temperature was kept constant. The interested reader may refer to the study of Martoïa et al. [19], who systematically investigated the role of freezing temperature. Thereafter, completely frozen samples from both routes (static and stirred freezing process) were thawed in denatured ethanol (95% ethanol, 5% isopropanol) at room temperature, with 4 cubes (132 mL total volume) thawed twice in beakers with 2 L of solvent. As a final step, the foams were dried directly in a ventilated oven at 65 °C for at least three hours, to ensure complete solvent evaporation.

Thawing in ethanol has multiple benefits including: (i) speed-up of the thawing process (~1 h), compared to air (>12 h in [24]), (ii) removal of urea, and (iii) exchange from water to ethanol (the residual water content after the second washing step is estimated to be <0.5%). Furthermore, ethanol has lower surface tension (*σ* = 22 mN/m) than water (*σ* = 73 mN/m). This reduces the capillary forces exerted by the evaporating meniscus on the cellulose structure, leading to a reduced amount of structural collapse during drying, compared to foams dried directly from water. Interestingly, we also noticed that even in the fully wet state, foams thawed in ethanol are stiffer than those thawed in water. Although this aspect was not specifically investigated by dedicated mechanical compression tests, it suggests that after their exchange to ethanol, the fibrils start to interact again and rebuild hydrogen bonds, thereby increasing the foam mechanical stability already in the thawed wet state, even before drying. The use of other solvents, such as isopropanol [27], can lead to similar results.

**Figure 3:**
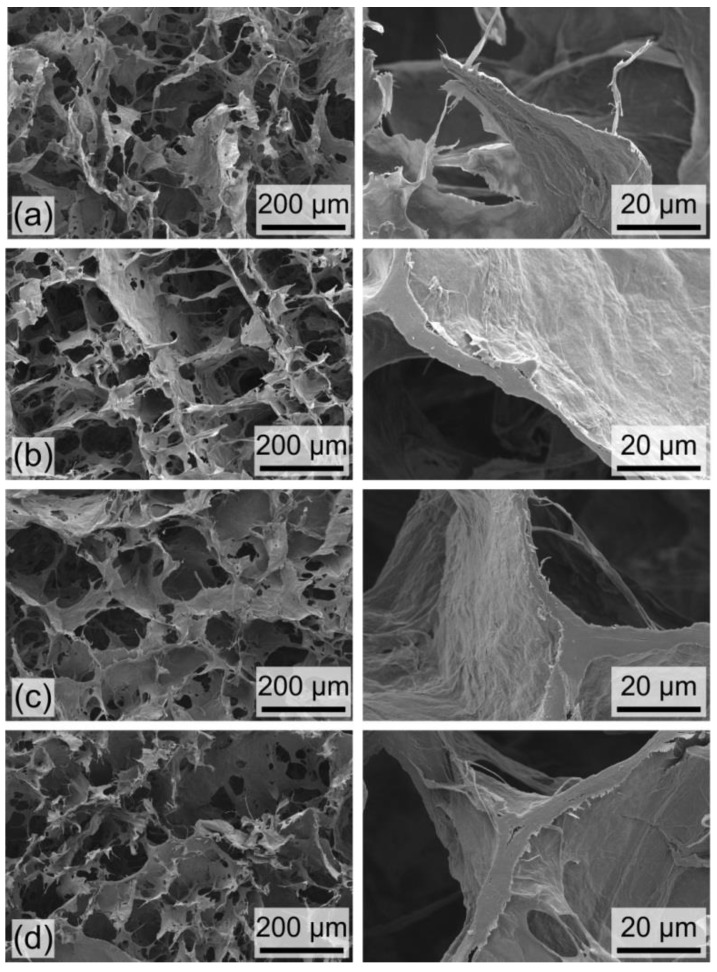
SEM images of cellulose foams produced via the stirred freezing process with different CNF concentrations: (**a**) 0.69 wt%, (**b**) 1.16 wt%, (**c**) 2.03 wt%, and (**d**) 2.39 wt%. Images in the left column highlight the characteristic porosity, with pore sizes in the order of 100 µm; images in the right column zoom in on representative wall thicknesses; these are in the range 1–10 µm and increases for increasing CNF concentration.

**Figure 4:**
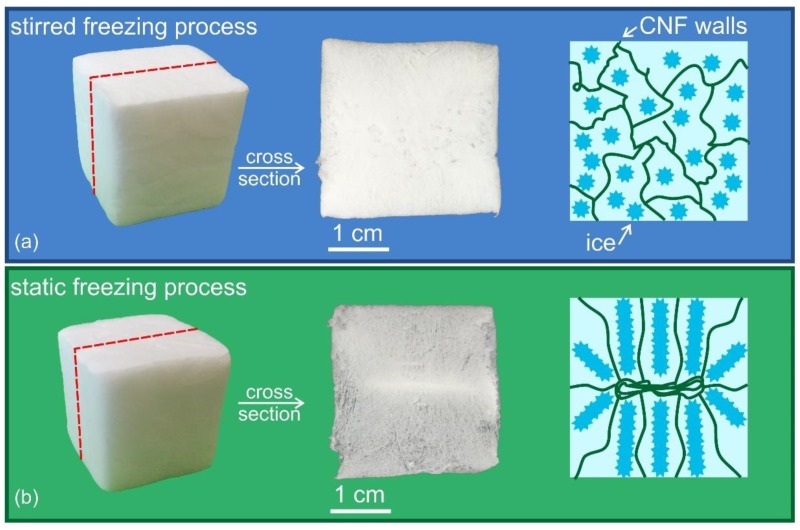
CNF foams produced via the stirred freezing process (**a**), with CNF concentration equal to 1.6 wt%, and foams formed by the static freezing process (**b**), with CNF concentration equal to 1 wt%. The images on the left are the final dry cubic foams and the center images show the sample cross sections. On the right side, schematic images highlight that, in the stirred freezing process, the stirring process leads to a more homogeneous distribution and orientation of ice crystal (blue stars) compared to the static freezing process, where a preferential growth of the ice crystals, from the external surface to the center of the sample, and a less homogeneous CNF (green lines) distribution is observed. Samples were cut using a razor blade for inspection of the cross section.

*Foam characterization.* The characteristic structure of the fabricated nanocellulose foams is visible in Figure 3 and Figure 4, including SEM and optical images of the foams.

The SEM images in Figure 3 highlight the characteristic porosity of the nanocellulose foams produced following the stirred freezing process for different CNF concentrations: (a) 0.69 wt%, (b) 1.16 wt%, (c) 2.03 wt%, and (d) 2.39 wt%. All foams possess a characteristic porosity imparted by ice-templating, with a pore size in the order of 100 µm (as visible by SEM in the left column in Figure 3), corresponding to the characteristic size of the formed ice crystals. A clear difference between the samples can be seen by the wall thickness. The thickness typically lies in the range 1–10 µm (right column in Figure 3) and increases to increase CNF concentration in the initial suspension.

Figure 4 highlights the different structures formed following the ice-cream and the static freezing processes. Although the samples appear similar on the outer surface (left column), a visual comparison of the cross-section (center column), provides an insight of the material isotropy: following the stirred freezing process, the sample develops an isotropic porosity distribution, whereas a clear anisotropic distribution is observed in the case of the static freezing process. In this case, the sample freezes statically in the silicone mold. As such, ice crystals grow from the outer surface to the core of the sample, creating a structure—and, thus, pores, once ice is removed—with a preferential orientation along the crystal growth (see schematic in the right column in Figure 4). Further, the central part of the sample appears denser, indicating that density is not homogeneous across the sample. Differently, for samples fabricated via the stirred freezing process, the preferential crystal growth is mitigated by the continuous stirring of the suspension in the first freezing step (see Figure 2). The stirred freezing process is thus beneficial since it can promote a more isotropic porous structure, in addition to potentially reducing the freezing time in a scaled-up process, due to the enhanced heat transfer in a stirred suspension.

To evaluate the foam characteristics in terms of porosity, density, and liquid absorption capacity, oil absorption tests were performed. Oil absorption was chosen as a method for two main reasons: on one hand, the test is rapid and enables the measurement of density and porosity even on samples that are not perfectly cubic, such as those that are subject to more severe shrinkage after the drying step (see details below). On the other hand, selective oil absorption is one of the potential applications of ultra-light foams, after the foam is appropriately hydrophobized.

**Figure 5:**
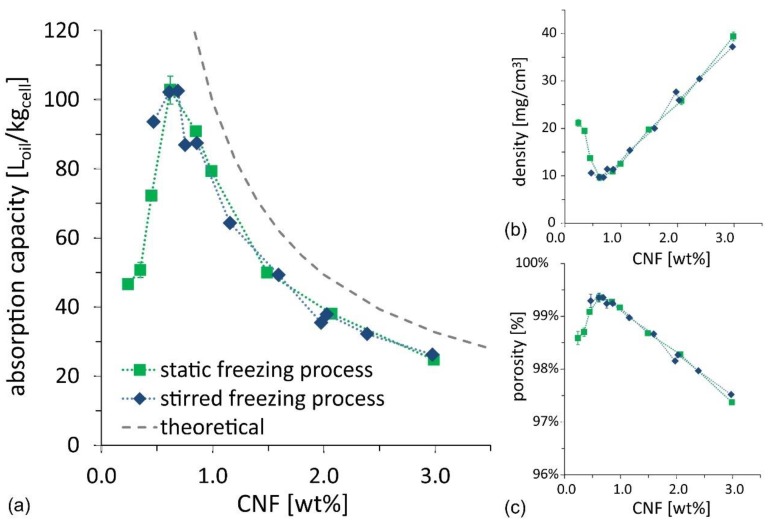
(**a**) Oil absorption capacity as a function of suspension concentration for CNF foams produced using the stirred freezing process (blue diamonds) and the static freezing process (green squares). The expected theoretical absorption capacity is also illustrated; deviation from theory is discussed in the text. (**b**) Density and (**c**) porosity as function of suspension concentration, for CNF foams produced using the compared processes. Lines connecting experimental data are provided to guide the eye.

The results of the oil absorption tests (see details of oil composition in the Methods and Materials section) are illustrated in Figure 5a as a function of CNF suspension concentration, for both the ice-cream and the static freezing process. The oil absorption capacity for samples from the two routes matches well and shows a clear non-monotonic trend, with an optimum value for the oil absorption capacity reaching 100 L_oil_/kg_cell_, for a CNF concentration of ~0.6–0.7 wt%, calculated as the ratio between the absorbed oil volume and the cellulose mass. Since the foams do not swell and preserve their shape when filled with oil, the absorption test outcome was also used to evaluate the density and porosity of dry foams. The values are illustrated as a function of CNF suspension concentration in Figure 5b,c. The results confirm that the optimum is achieved at CNF concentration of ~0.6–0.7 wt%, for which density is minimized to 10 mg/cm^3^, and porosity is maximized to 99.4%. The optimum is a result of two competing requirements: (i) The CNF concentration in the initial suspension should be as low as possible to reduce cellulose mass and thus to increase the available porosity; and (ii) the CNF concentration needs to be high enough to provide a self-sustained CNF skeleton that prevents the foams from shrinking upon drying. The former requirement explains the increasing oil absorption capacity, found when decreasing CNF concentration in the high concentration regime, and the latter requirement explains the sudden drop in oil absorption capacity in the low concentration regime.

**Figure 6:**
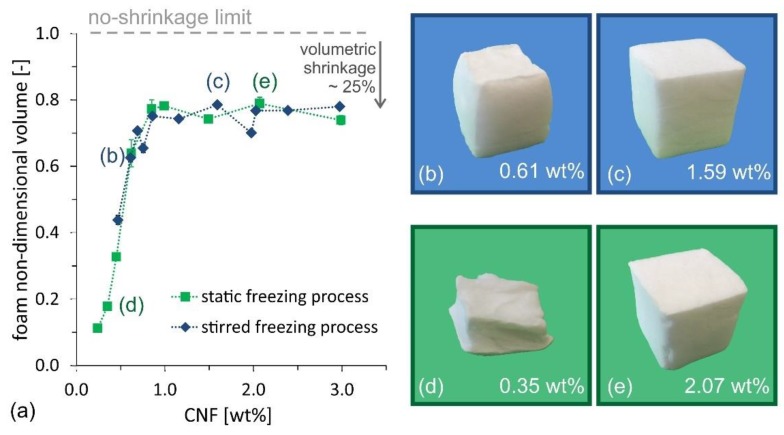
(**a**) Foam non-dimensional volume, calculated as the ratio between the final volume of the foam and the initial suspension volume, as function of suspension concentration, for CNF foams produced using the stirred freezing process (blue diamonds) and the static freezing process (green squares). Lines connecting experimental data are provided to guide the eye. Pictures illustrate representative samples produced using the stirred freezing process (**b**,**c**), with CNF concentration equal to 0.61 wt% and 1.59 wt%, respectively) and the static freezing process (**d**,**e**), with a CNF concentration equal to 0.35 wt% and 2.07 wt%, respectively).

The competition between the two requirements becomes even clearer when looking at the evolution of the dry foam volume, made non-dimensional with the initial suspension volume (33 mL) as a function of the CNF suspension concentration (see Figure 6a). At high CNF concentration, all samples experience shrinkage upon drying, with ~25% volume reduction compared to the initial cube volume, whose value corresponds to an isotropic linear shrinkage of ~10%. However, for concentrations below 0.8 wt%, the volume shrinkage increases sharply (i.e., the foam collapses upon drying). The optimum condition for oil absorption capacity, identified in the range 0.6–0.7 wt%, can be approximated in Figure 6a, as the maximum ratio between foam non-dimensional volume and CNF concentration. The partial foam shrinkage during the drying process, observed even at concentrations higher than 0.7 wt%, also explains why the experimentally measured values for oil absorption, in Figure 5, are lower than the theoretical values, which are calculated for the ideal case of zero-shrinkage.

With respect to porosity and density values, Li et al. [27] recently reported the production of high porosity nanocellulose foams with a porosity of ~98% and a density of 18 mg/cm^3^. Thus, with the process described here, we could reduce the density value by a factor of 2. Further, foam density values of 10 mg/cm^3^ compete well with the best reported values in the literature for nanocellulose foams produced by freeze-drying, based on vacuum drying, reporting values of 60 mg/cm^3^ [21], 14 mg/cm^3^ [7], 12.5 mg/cm^3^ [19], 6.7 mg/cm^3^ [12], and 5.6 mg/cm^3^ [8], respectively.

Finally, sorption tests were performed using krypton and nitrogen. For CNF concentrations in the range 0.69 wt% to 2.98 wt%, specific surface area values were 10–15 m^2^/g by krypton sorption and 4–10 m^2^/g by nitrogen sorption. These values are comparable to other works, reporting values of the same order of magnitude [27,28].

**Figure 7:**
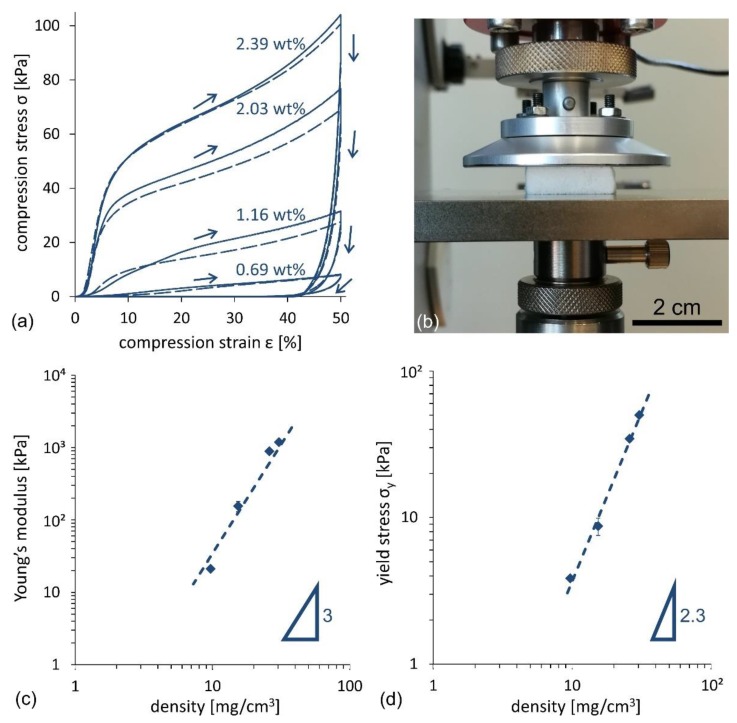
Mechanical compression tests for CNF foams produced by the stirred freezing process. (**a**) Compression stress as a function of compression strain up to 50%. The numbers refer to CNF suspension concentrations of 0.69 wt%, 1.16 wt%, 2.03 wt%, and 2.39 wt%. Two lines (solid and dashed) refer to two different tests on two different samples. (**b**) Picture of the experimental setup. (**c**,**d**) Evaluation of the Young’s modulus (**c**) and the yield stress (**d**) as a function of foam density.

The mechanical properties of CNF foams, produced following the stirred freezing process, were tested on 20 × 20 × 10 mm^3^ samples. The main findings are reported in Figure 7. Figure 7a illustrates the compression stress–strain *σ–ε* curve for four different samples (see setup in Figure 7b) obtained from the CNF suspension concentrations, ranging from 0.69 wt% to 2.39 wt%. All samples denote a characteristic compression curve. After the initial yield, the curves show a linear part, characteristic of the elastic region, followed by a nearly linear plastic deformation region above the yield stress. Following the guidelines in [19,29], the values of the Young’s modulus, *E*, and the yield stress, *σ*_Y_, as a function of the foam density, were derived and are illustrated in Figure 7c,d, respectively (see the Methods and Materials section for further details). The order of magnitude of Young’s moduli ranges from 10 to 10^3^ kPa, where the observed scaling is $E\propto \rho_{\mathrm{foam}}^{n}$, with *n* *≈ 3*. Young’s modulus values are similar to those of the freeze-dried CNF foams, as summarized by Donius et al. [30], who reported a comprehensive comparison of mechanical properties for a variety of cellulose foams and aerogels. The yield stress values span from 4 to 40 kPa, where a scaling $\sigma_{Y}\propto \rho_{foam}^{n}$, with *n* *≈ 2.3*, was found, in agreement with [19].

**Figure 8:**
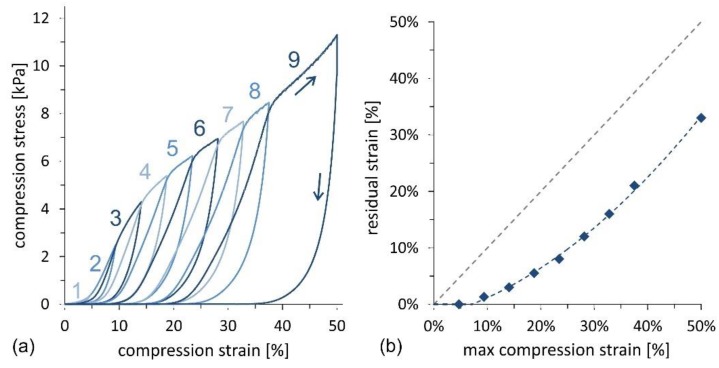
Mechanical compression tests of CNF foams produced by the stirred freezing process and a CNF concentration equal to 0.69 wt%. (**a**) Compression stress as function of compression strain including 9 cycles at increasing maximum compression strain up to 50%. The number beside each curve denote the cycle number. (**b**) Residual strain as function of the maximum compression strain as extracted from the data in (**a**). The limit for the elastic behavior, corresponding to zero residual stress, is found for a maximum compression strain of ~8%. The fitting line is a polynomial curve, while the dashed gray line is the quadrant bisector.

Compression cycles with an increasing maximum strain, up to 50%, were also performed to investigate the mechanical response of the material in more detail. Figure 8a illustrates the σ–ε curve for the sample with the highest oil absorption capacity, corresponding to a CNF concentration of 0.69 wt% and fabricated following the stirred freezing process. Incremental loads enable evaluation of the residual strain as a function of the maximum compression strain for each cycle, as illustrated in Figure 8b. Fitting of experimental data shows that the limit for elastic behavior, characterized by zero residual strain, is found at ~8% maximum strain. Below this compression threshold, the foam expands back to the original thickness after compression, whereas above the threshold, a plastic deformation behavior is observed.

As an outlook for future studies, we foresee the need to further investigate the interaction of urea with the CNF suspension during the process. Urea may interact either directly with cellulose or non-cellulosic components (such as hemicellulose and lignin) [31] or may affect the nucleation process.

**Figure 9:**
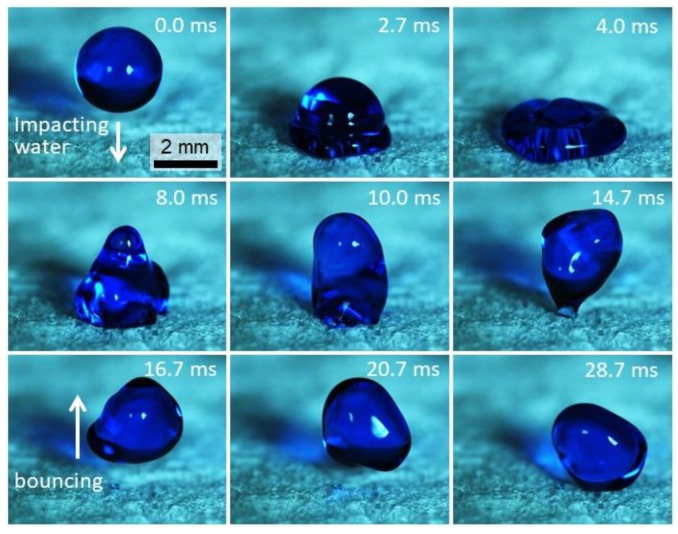
High-speed image sequence of a water drop impacting and rebounding from CNF foams. Impact conditions: drop diameter *D* = 2.71 mm, impact velocity *V* = 0.39 m/s. Water is blue-stained to improve contrast. The corresponding time for each frame is indicated.

To impart hydrophobicity, foams were functionalized using alkylketenedimer (AKD) (more specifically the commercially available product Aquapel F210) an organic compound used in the paper industry to reduce hydrophilicity. After preparation, cellulose foams were immersed in a suspension of AKD and ethanol and re-dried (diluted to 1:128 wt%). As visible in the image sequence in Figure 9 (and corresponding Supplementary Video SV1), captured by high-speed imaging (1500 frames per second), foams functionalized by AKD became superhydrophobic. As such, water drops were able to rebound after impact on the surface of the nanocellulose foam, as typically observed on superhydrophobic surfaces [32,33].

**Figure 10:**
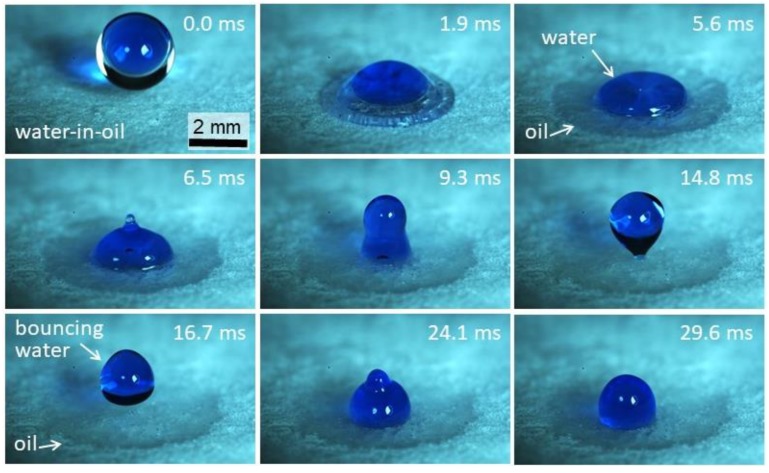
High-speed image sequence of a compound water-in-oil (i.e., dodecane) drop impacting on CNF foams. Impact conditions: drop diameter *D* = 2.65 mm, impact velocity *V* = 1.01 m/s, and water-to-oil ratio α = 0.3. Water is blue-stained to improve contrast. Corresponding time for each frame is indicated.

As such, nanocellulose foams have the potential to be used to separate water–oil emulsions. In our previous paper, we showed that when superhydrophobic porous cellulose films were exposed to a nebulized mixture of 50:50 vol:vol dodecane and water, water drops coalesced and slid down the film surface, whereas dodecane penetrated into the film and was retained [28]. Using high-speed imaging, here we show the details of the separation mechanism, which is visualized in the image sequence in Figure 10 (and corresponding Supplementary Video SV2), where the impact of a compound water-in-oil drop is captured. After the initial inertia-driven spreading of the liquids, the oil keeps wetting and becomes imbibed by the oleophilic foam, without any recoil. Conversely, the water core undergoes recoil and rebounds after impact, due to the combined effect of substrate superhydrophobicity and lubricating oil layer. Note that when the bouncing water drop escapes, we expect it to remain covered by a film of oil, since the sum of interfacial water–oil tension for the investigated system (*σ*_ow_ = 53 mN/m) [34], and the oil surface tension, *σ*_o_, is approximately the same as water surface tension: *σ*_ow_ + *σ*_o_ ≈ *σ*_w_. The mechanism for separation of water and oil mixtures shown here will be the focus of a more systematic study, focusing on the conditions for water core rebound, as well as drop shedding by shear forces, such as gravity and aerodynamic forces.

## 3. Conclusions

We have reported here the development of an optimized fabrication process for highly porous cellulose foams, based on a well-controlled freeze-thawing-drying (FTD) process at ambient pressure. The process enables fabrication of foams with extreme porosity, up to 99.4%, a low density of 10 mg/cm^3^, and an oil absorption capacity of 100 L/kg, with optimal results achieved for a suspension concentration in the range 0.6–0.7 wt% for the nanocellulose used in the present study, having a specific surface area of 200 m^2^/g. The optimal CNF suspension concentration is found as the compromise between two competing requirements: (i) a concentration as low as possible, to reduce cellulose mass and increase porosity; and (ii) a high enough concentration to create a self-sustained CNF skeleton and avoid foam collapse upon drying. Foams were characterized by means of mechanical tests, which showed Young’s moduli in the range 10–10^3^ kPa and yield stress values in the range 4–40 kPa. Finally, we show how a commonly used AKD-based hydrophobizing agent can be used to impart superhydrophobicity and how foams can be used to separate water-in-oil compound drops via drop impact on the nanocellulose foams. As such, we demonstrate the potential of nanocellulose foams to separate immiscible liquids for application in the fields of environmental remediation or liquid purification.

## 4. Methods and Materials

*Materials.* CNF suspensions were produced from cellulose dry boards (Eucalyptus pulp), obtained from the company Schattdecor AG (Thansau, Germany). Cellulose boards were chopped up and swollen in water at a concentration of 1.42 wt% and subsequently ground with an ultra-fine friction grinder ‘‘Supermasscolloider’’ (MKZA10-20J CE, Masuko Sangyo Co., Ltd., Kawaguchi/Saitama, Japan) [24,35]. A grinding energy input of 9 kWh/kg referred to dry CNF content was applied. After grinding, a part of the CNF suspension was dewatered on a sieve under pressure to reach a solid content of about 5.7 wt% for preparation of foams at higher CNF concentration. The cellulose pulp was not chemically treated (i.e., non-oxidized) before mechanical disintegration.

*Scanning electron microscopy.* Scanning electron microscopy (SEM) was performed on both cellulose nanofibrils and foams using a Fei Nova Nanosem 230 Instrument (Fei, Hillsboro, OR, USA). To minimize nanofibril aggregation upon drying, CNF suspension was diluted to 0.05 wt%, and a sample drop was placed on a mica wafer, dried inside the sputter device under high vacuum, and finally sputtered with 7 nm platinum (BAL-TEC MED 020 Modular High Vacuum Coating Systems, 117 BAL-TEC AG, Balzers, Liechtenstein) to enhance sample electrical conductivity.

*Specific surface area.* The specific surface area (SSA) was determined by means of the Brunauer-Emmett-Teller (BET) method, both on supercritically dried cellulose nanofibrils and on foams [36]. Nanofibrils were super-critically dried (Quorum Technologies E3100, Laughton, UK), performing solvent exchange from water to liquid CO_2_, via ethanol (10 °C and 50 bar), followed by drying at supercritical conditions (35 °C and 100 bar). Dried nanofibrils were then degassed at 105 °C for 4 h, before performing nitrogen sorption measurement (SA3100, Beckman Coulter, Indianapolis, IN, USA).

For foams, sorption was performed with a Micromeritics 3 Flex (Micromeritics, Norcross, GA, USA) using both krypton and nitrogen. Foams were degassed at 105 °C in ultimate vacuum of 2 × 10^−3^ mmHg for 16 h prior to the measurements.

*Oil absorption tests.* Foams were characterized by oil absorption tests, to assess the foam liquid absorption capability, as well as to determine foam density and porosity. As a liquid, we used a mineral oil prepared by the company Weidmann Electrical Technology AG (Rapperswil-Jona, Switzerland), which is intended to simulate diesel fuel and domestic heating oils. This oil is composed of a mixture of straight-chain paraffins and 1-methylnaphtalene. The mineral oil was characterized in terms of density (*ρ*_oil_ = 828 ± 4 kg/m^3^) and surface tension (*σ*_oil_ = 23.0 ± 0.3 mN/m) and used as received. For oil absorption tests, foams were initially prepared by drying in the oven at 65 °C for at least one hour before tests, to ensure complete desorption of water on the cellulose surface, which may accumulate due to environmental humidity (typical water adsorption on foam is 5%–8% when stored in the laboratory). The foam dry mass (*m*_dry_) was first measured. Subsequently, the foam was gently placed in a beaker containing at least 100 mL oil and kept submerged for at least 30 s and then gently lifted using tweezers and placed on the balance, to measure the total mass uptake, $m_{\mathrm{uptake}}=m_{\mathrm{dry}}+m_{\mathrm{oil}}$. The oil absorption capacity, *C*, was subsequently calculated as $C=V_{\mathrm{oil}}/m_{\mathrm{dry}}$, where the volume *V*_oil_ is derived from the oil density, *ρ*_oil_. The volume is used, to express oil absorption capacity independently from oil density (otherwise, using the mass ratio, denser oil would give higher values than lower density oil).

*Foam density and porosity calculation.* Since the foams do not swell during oil absorption, oil capacity values were used to estimate both density and porosity. The estimation is based on the assumption that negligible bubbles remain trapped in the foam, which is confirmed by the fact that the imbibed foams sink in oil and do not float. Also, the assumption is conservative, since the undesirable presence of bubbles would cause lower absorption of mineral oil, and thus lead to an underestimation of porosity and overestimation of foam density.

*Mechanical tests.* For each dry CNF foam, compression tests were performed on 4 samples, prepared by sawing, with dimensions of 20 × 20 × 10 mm^3^. Compression tests were performed using a universal testing machine type Zwick 1484 (Zwick GmbH & Co. KG, Ulm, Germany), equipped with a 500 N load cell and crosshead displacement sensor, both with an accuracy of measurement of 1% (class I). The compression speed in the vertical (z) direction was 1 mm/min, down to a 50% compressive strain. Two samples were compressed and decompressed in a single cycle, and two samples were compressed with an increasing maximum strain (9 cycles up to 50%), to evaluate the residual strain under different maximum compression strains. Evaluation of the Young’s modulus, *E*, and the yield stress, *σ_Y_*, was performed as described in [19] and based on [29]. The Young’s modulus *E* was estimated from the initial linear part of the *σ–ε* curve, and the yield stress, *σ_Y_*, was calculated from the intersection of the two tangent lines parallel to the apparent elastic domain and the nearly linear strain hardening plasticity regime above the yield stress.

*Foam hydrophobization.* Alkylketenedimer (AKD), commercial name Aquapel F210, was donated by Solenis, Switzerland. AKD was diluted in ethanol (1:128 wt%) and ultrasonicated for 15 min to promote a homogeneous dispersion. For drop impact tests, nanocellulose foam plates (200 × 200 mm^2^, and thickness of 20–30 mm) were immersed in the AKD-ethanol dispersion and re-dried in the oven at 65 °C.

*Drop impact experiments.* The compound drop was generated with a coaxial needle connected to a Harvard Apparatus with two syringe pumps (PHD 2000 and Pico Plus Blue). The coaxial needle contained an inner needle (inner diameter: 0.16 ± 0.019 mm; outer diameter: 0.31 ± 0.064 mm) providing pure water (Milli-Q system) and an outer needle (inner diameter: 0.55 ± 0.013 mm; outer diameter: 0.80 ± 0.038 mm) providing oil, i.e., anhydrous dodecane, ~99% (Sigma Aldrich, Buchs, Switzerland, *ρ*_d_ = 750 kg/m^3^, *μ* = 1.34 mPa s at 25 °C). To enhance contrast in optical images and visualize oil absorption and water repellency, water was stained blue by adding Methylene Blue (Sigma Aldrich, Shangai, China, density 1000 kg/m^3^) at concentration of 0.06%, while the oil remained colorless.

The optical equipment consisted of a high-speed camera (color Photron FASTCAM Mini WX100) recording the drop impact event at 1500 fps (shutter speed of 1/12,000 s, resolution 2048 pixels × 1472 pixels). To allow a better view of the impact sequence, the camera, equipped with a macro lens (Leica Z16APO), was tilted with an angle of 25°. Two light sources (Sumita LS-M352) were used.

Before the impact, a series of drops were collected in a container until the water-to-oil ratio of the compound drop (α = 0.3) stabilized with a quasi-steady pinch-off. Note that, due to surface irregularities (such as roughness and waving), impacts showed differences in outcomes over the 90 tests performed.

## Supplementary Materials

The following are available online at https://www.mdpi.com/2079-4991/9/8/1142/s1, CNF-urea parameter space, two supplemental videos of drop impact on the CNF foam.
