# Supplementary material for: Ultra-Porous Nanocellulose Foams: A Facile and Scalable Fabrication Approach
Source: Nanomaterials (Basel). 2019 Aug 9;9(8):1142. doi: 10.3390/nano9081142 (PMC6723185; doi:10.3390/nano9081142)
Supplement: Supplementary file 1 [file nanomaterials-09-01142-s001.zip › Cellulose_foam_oil_SI_REV_RESUB.pdf]

Supplemental Information to

"Ultra-porous nanocellulose foams: a facile and scalable fabrication approach"

Carlo Antonini[1,2], Tingting Wu[1], Tanja Zimmermann[1], Abderrahmane Kherbeche[3], Marie-Jean Thoraval[3], Gustav Nyström[1], Thomas Geiger[1]

[1] Cellulose and Wood Materials, Swiss Federal Laboratories for Materials Science and Technology (Empa), Dübendorf, Switzerland

[2] Department of Materials Science, University of Milano - Bicocca, Milano, Italy

[3] State Key Laboratory for Strength and Vibration of Mechanical Structures, Shaanxi Key Laboratory of Environment and Control for Flight Vehicle, International Center for Applied Mechanics, School of Aerospace, Xi'an Jiaotong University, Xi'an 710049, P. R. China

Corresponding authors: carlo.antonini@unimib.it, thomas.geiger@empa.ch

1. OPTIMIZATION OF THE CNF-UREA RATIO

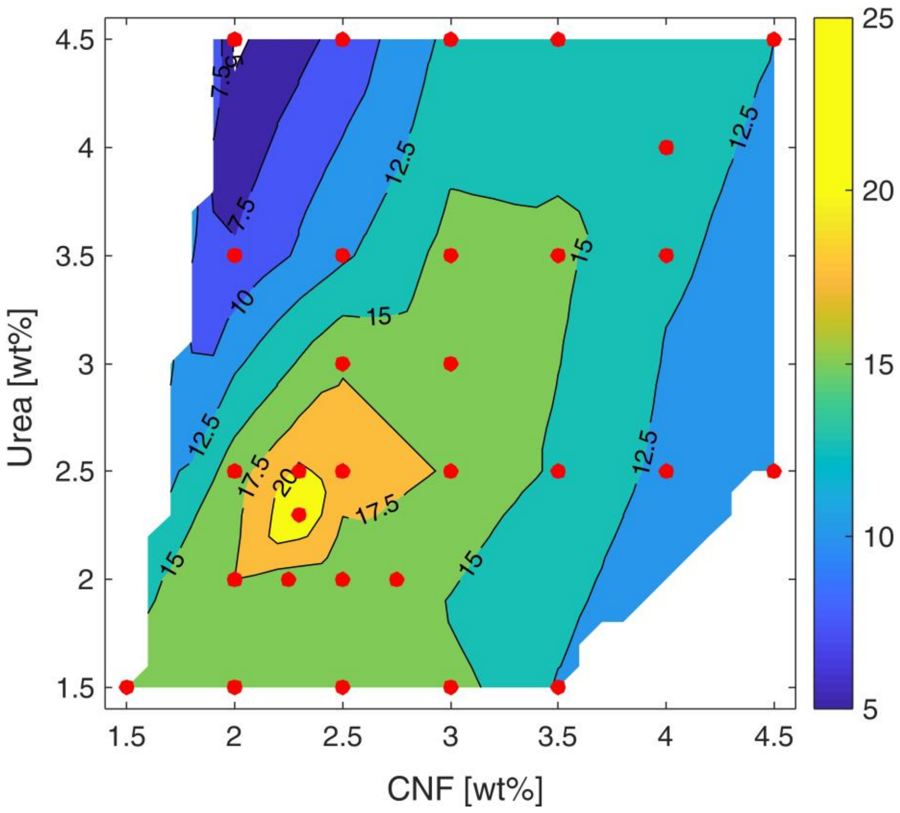

Figure S1: CNF-urea parameter space showing the oil absorption capacity, in case of thawing in water (instead of ethanol). Preliminary optimization tests show that a ratio 1:1 of CNF-urea maximizes oil absorption. Red dots correspond to experimentally tested combinations. Contour plot was created by data interpolation within the experimentally tested conditions. Absolute maximum of oil absorption (22 L_{oil}/kg_{cell}) was obtained for CNF and urea equal to 2.3 wt%. Tests were performed using the commercially available product MFC Advanced, provided by Weidmann Electrical Technology AG.

Preliminary tests were performed to optimize the CNF-urea ratio, with the goal of maximizing foam absorption capacity. Tests were performed using CNF supplied by the company Weidmann Electrical Technology AG (Switzerland). We used the material commercially available as “MFC Advanced”. Foams were produced using the stirred freezing process described in the main text, with the only difference that thawing was performed in water and not in ethanol. The values of oil absorption capacity are illustrated in Figure S1, as function of CNF concentration (ranging from

1.5 to 4.5 wt%) and urea concentration (1.5 to 5 wt%). Water-thawed foams show overall lower absolute values of oil absorption capacity, with values up to 22 L_{oil}/kg_{cell}. Nonetheless, a relative comparison of absorption capacity is valuable. Indeed, we found out that optimal results were obtained for 1:1 ratio of CNF-urea; highest oil absorption capacities were obtained for concentrations of CNF and urea equal to 2.3 wt%.

2. DETERMINATION OF FOAM MECHANICAL PROPERTIES

Figure S2 illustrates how the Young's modulus and the yield stress of foams were determined from experimental σ-ε curves. The Young's modulus was calculated as the slope of initial linear part of the σ-ε curves, typically for $\varepsilon < 5\%$. The apparent yield stress, σ_y, was calculated from the intersection of the two tangent lines parallel to the elastic linear domain and the nearly linear strain hardening plasticity regime above the yield stress

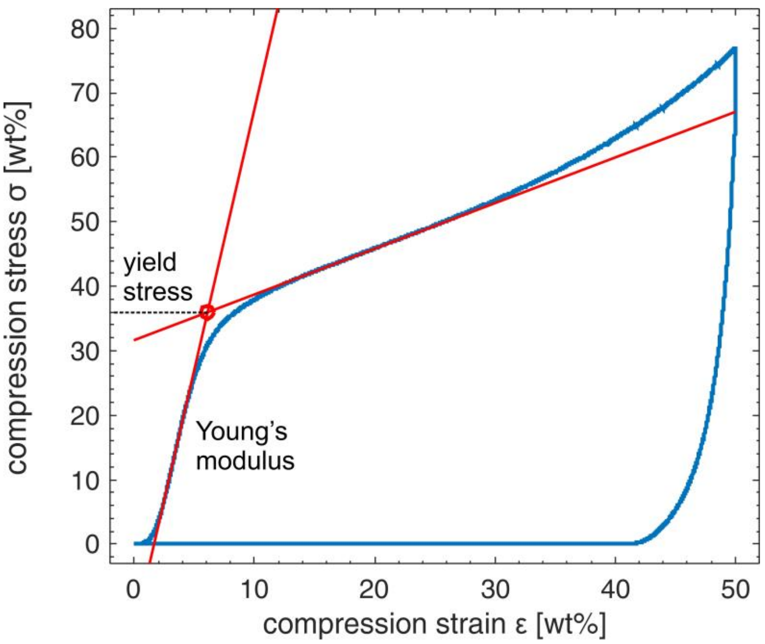

Figure S2: Evaluation of Young's modulus and yield stress from mechanical compression tests.
